# Preparation of antimicrobial polymeric composites using defective silk cocoons and moringa seed oil as additives for polyvinyl chloride

**DOI:** 10.1038/s41598-025-97540-z

**Published:** 2025-05-05

**Authors:** Nagwa A. kamel, Nehad N. Rozik, Salwa L. Abd El-Messieh

**Affiliations:** 1 Microwave Physics and Dielectrics Department, Physics Research Institute. National Research Centre, Dokki, Cairo, Egypt; 2 Polymers and Pigments Department, Chemical Industrial Research Institute. National Research Centre, Dokki, Cairo, Egypt

**Keywords:** Bio-based plasticizer, PVC composites, Silk cocoons, Antimicrobial, Dielectric, Biophysics, Materials science

## Abstract

In this work, novel polymeric blends were prepared from polyvinyl chloride (PVC) and silkworm cocoon waste (SCW), that were defective cocoons excluded during the silk-making process in the ratio 50:50 w/w. These blends were incorporated with moringa seed oil (MSO) as a bio-based plasticizer with different concentrations (1, 2, and 3%) to obtain a final bioplastic with superior antimicrobial properties. The new composites are characterized through Scanning Electron Microscope (SEM), Fourier Transmission Infrared Spectroscopy (FTIR), contact angle measurements, Thermogravimetric analysis (TGA), dielectric, mechanical, and antimicrobial properties. Results of the study pointed to improved linking between the blend phases after incorporating 2% MSO. The composites could inhibit the growth of all the tested microorganisms. The conductivity σ_dc_ values increased by increasing the content of MSO in the composite. The results demonstrate the potential of the new MSO plasticized composites as promising candidates for use in hospitals as antimicrobial surfaces.

## Introduction

One of the primary causes of death in hospitals worldwide is the bacterial infections. Bacteria may grow on a wide variety of surfaces, and when they do, biofilms are created as the bacteria colonize the surface^1^. PVC is a plastic that may be used for everyday purposes and is inexpensive, strong, and recyclable^2^. PVC has several applications, which create an imaginative challenge.

They surround us daily: credit cards, roofing membranes, medical equipment, gas and water pipelines, construction profiles, and kids’toys. Although polyvinyl chloride (PVC) is frequently utilized to make hospital equipment and biomedical devices, it lacks sufficient antibacterial action to stop biofouling^3^. In health care, hospital privacy curtains usually made from PVC, are constantly touched but rarely changed, making them a prime candidate for cross-contamination^4^. The development of antimicrobial PVC sheets for medical environments is essential.

The most frequently used type of PVC plasticizer is conventional petrochemical-based phthalate plasticizer, such as diethylhexyl phthalates (DEHP) and diisononyl phthalates (DINP). However, several concerns about the use of phthalates have lately been brought forth^5^. Numerous studies have documented the use of vegetable oils as alternative plasticizers for PVC^6–8^.

Lim KM et al. investigated the influence of adding the alkyd based on palm oil as a co-plasticizer to di-isononyl phthalate and di-octyl phthalate on the morphological, mechanical, and thermal properties of PVC. The outcomes showed that adding the alkyd improved the PVC’s characteristics^9^.

The plant Moringa oleifera, which goes by several names, including “drumstick tree” or “horseradish tree,” has gained popularity throughout the world for its nutritional and medicinal qualities^10^. The most prevalent species in the Moringa genus is Moringa oleifera, which has antimicrobial properties and is a rich source of several phytochemical substances, including glucosinolates and high levels of stable oleic acids. Amina, Musarat, et al. Fabricated antimicrobial nanocomposite from PVC enriched with moringa oil and silver nanoparticles. The results confirmed the film’s effectiveness against the tested microorganisms^11^.

Sericulture is the technique of raising silkworms and harvesting silk from them. It is a major local industry in several countries. Usually, there are defective cocoons ,such as cocoons with loose shells, molded cocoons, melted cocoons, and damaged cocoons. Defective cocoons negatively impact raw silk purity, dependability, and proportion.

Silkworm cocoons have antibacterial qualities that can shield them from germs and guarantee the pupae have a smooth growth phase^12^. Because of the nature of this waste, it can’t be employed in the traditional textile industry. Defective cocoons are one type of this waste. It is of great power in research due to its fibroin content. Roughly 11 million tons of silk waste are produced annually worldwide, accounting for about 35% of all silk produced worldwide^13^. Jarupong et al. used cocoon waste as a reinforcer in the epoxy resin matrix composites. The results confirmed the improvement of the mechanical properties^14^. Jeerasak Jarupong et al. produced composite plates employing waste silkworm cocoon-reinforced polymer composite and epoxy resin matrix to replace synthetic fibres in bulletproof plates. Silkworm cocoon waste was significant as a reinforcing agent compared to hemp-woven materials^15^.

The goal of this study is the preparation of new super antimicrobial films by a combination of PVC with treated defective silk cocoon waste by the ratio 50:50 w/w and replace the traditional harmful plasticizers of PVC with Moringa oleifera seed oil as green plasticizer and antimicrobial agent, theses superior antimicrobial composites aimed to utilized in hospitals as privacy curtains to replace the conventional textiles to control infections.

## Materials and methods

### Materials

Silkworm cocoons produced by Bombyx mori silkworms were used as row material. Formic acid (CH_2_O_2_, ≥ 98%, Sigma-Aldrich, Germany) and lithium bromide (LiBr, ≥ 99%) were used exactly as supplied, requiring no purification. Polyvinyl chloride (PVC) produced using suspension polymerization with a k value of 67 supplied from El-Amerria (Alexandria, Egypt).

Moringa seed oil was purchased from the association of moringa-agricultural unit- National Research Centre.

On dry surfaces like privacy curtains, some of the most prevalent hospital-acquired infections can persist for several months. Along with many gram-negative species like Escherichia coli and Pseudomonas aeruginosa, the majority of gram-positive bacteria, including Vancomycin Resistant Enterococcus (VRE), Staphylococcus aureus, including Methicillin Resistant Staphylococcus aureus (MRSA), and Streptococcus pyogenes, can survive for months on dry surfaces as can fungal pathogens such like Candida albicans^16^.

In this study, *Staphylococcus aureus* (ATCC 6538) is a type of Gram-positive bacteria, *Escherichia coli* (ATCC 25,922) as a type of Gram-negative bacteria, and Candida albicans as pathogenic yeast were chosen as pathogenic microorganisms to evaluate the antimicrobial effect of the new composites. The inoculation is made from fresh overnight broth cultures using nutrient broth medium. After adjusting the inoculum, the plates and flasks received 1.5 × 10^8^ CFU/mL of the fungus and both types of bacteria. The subsequent process outlines a way to prepare the intended inoculum by comparing it to a 0.5 McFarland standard^17^.

## Methods

### Silk cocoons treatment

The standard degumming method was used to extract silk sericin^18^. It included boiling 2.5 g of chopped silk cocoons per liter of 0.02 M sodium carbonate solution for 30 min. After that, the cocoons were dissolved in 9.3 M lithium bromide for four hours in an oven set at 60 °C. This procedure calls for solvents that can interact more strongly with the polar groups in the silk fibroin as an ionic liquid. As a result, the packed structures can open, and the chain becomes soluble. As previously noted, methylimidazole was used to prepare the ionic liquid (IL)^19^.

### Preparation of PVC/SCW blend films

SCW and PVC were blended at blending ratios of 50:50 wt/wt in the presence of 10% Ionic liquid. Following a one-hour stirring period, the mixture was transferred onto Petri plates with membrane casting, and the solvent was gradually removed in ambient conditions. The resultant films were dried for 48 h at 37 °C.

### Preparation of Moringa seed oil plasticized films

The PVC/SCW/MSO were fabricated by dissolving PVC/SCW 50:50 wt/wt polymer powder in Tetra hydro furan solution (THF). The mixture was stirred to form a homogeneous solution. Then, different percentages of MSO (1,2 and 3%) were added to the homogeneous solution using a magnetic stirrer. The solutions were transferred onto Petri plates and the resultant films were dried at room temperature. The thickness of the samples = 1.2 mm.

### Fourier-transform infrared (FTIR)

A Tokyo, Japan-based JASCO FT/IR 300 E FTIR Spectrometer was used to record the infrared spectrum. Number of scans:100 and the spectral range (4000–400 cm^–1^).

### Contact angle measurements

The sample’s contact angles were measured using a camera attached to a computer. At 25 °C, a little droplet of DI water (10 μL) was gently applied to the sample surface. The angle formed by the water–air and membrane-water interfaces were measured. Images were taken five seconds after the drip was introduced, and the contact angles were computed by image analysis. Each droplet was photographed at least ten times, and around five measurements were taken at various locations of the membrane sample. Every result is an average of five measurements. Standard deviations of the data were calculated^20^.

### Scanning electron microscope (SEM)

The surfaces of the samples were examined with a Philips XL30, Japan, SEM/EDX with an accelerating voltage of 30 kV. at magnification 3000X.

### Thermogravimetric analysis (TGA)

The thermal analysis performed using LINSEIS STA 1600. The powdered sample was heated by 20 °C/min up to500 °C in a nitrogen environment.

### Dielectric measurements

At room temperature (~ 30 °C), a wide frequency range (100 Hz–1 MHz) was used to evaluate permittivity ε', loss factor tan δ, and ac-resistance Rac. The broadband impedance analyzer (Schlumberger Solartron1260) was connected to a personal computer via a GPIB cable (IEEE488) to computerize the test. The data collecting software utilized was “Lab VIEW,” a commercial interface and automation package. Specifically, the inaccuracy in ε' and tan δ is ± 1% and ± 3%, respectively.

### Mechanical properties

According to ASTM D412-15a, tensile strength (MPa) and elongation at break (%) were determined for the composites using Zwick Z010 testing equipment (Germany). Dumbbell-shaped samples were created by cutting the molded sheets with a Wallace die cutter at a crosshead speed of 500 mm/min. The experiments were conducted at room temperature.

### Microbiological analysis

The shake flask method was utilized to perform the antimicrobial assay of the composites. This method determined the reduction of colony forming (CFU) by measuring the optical density OD at 600 nm. The inoculum size of these pathogenic strains was prepared from the fresh working stoke cultures and adjusted to approximately 0.5 McFarland standard (1.5 × 10^8^ CFU/mL), 1 disc from each sample was applied separately on these inoculated flasks, and both the bacterial and fungal suspensions (25.0 μL) were separately transferred under aseptic conditions into 100.0 conical flasks containing 20.0 ml of nutrient broth medium (NB). These flasks were then incubated for 24 h at 37 °C with 150.0 rpm rotating shaking conditions^21,21^^23,24^.

The antimicrobial activity was measured throughout the relative (OD %) reduction of these pathogenic strains in flasks holds the treated samples compared to the control flasks that contains pathogenic strains only without any treatments. The results were represented using the following formula:

1 $${\text{Relative}}\left[ {{\text{CFU}}\,{\text{Reduction }}\left( \% \right)} \right] = \left( {{\text{A}} - {\text{B}}} \right)/{\text{A}} \times {1}00$$

where A: The CFU of pathogenic strains alone, on a control flask without any treatments. B: The CFU of pathogenic strains existing on the flask contains the treated sample.

## Results and discussion

### Surface color and appearance of the films

**Fig. 1:**
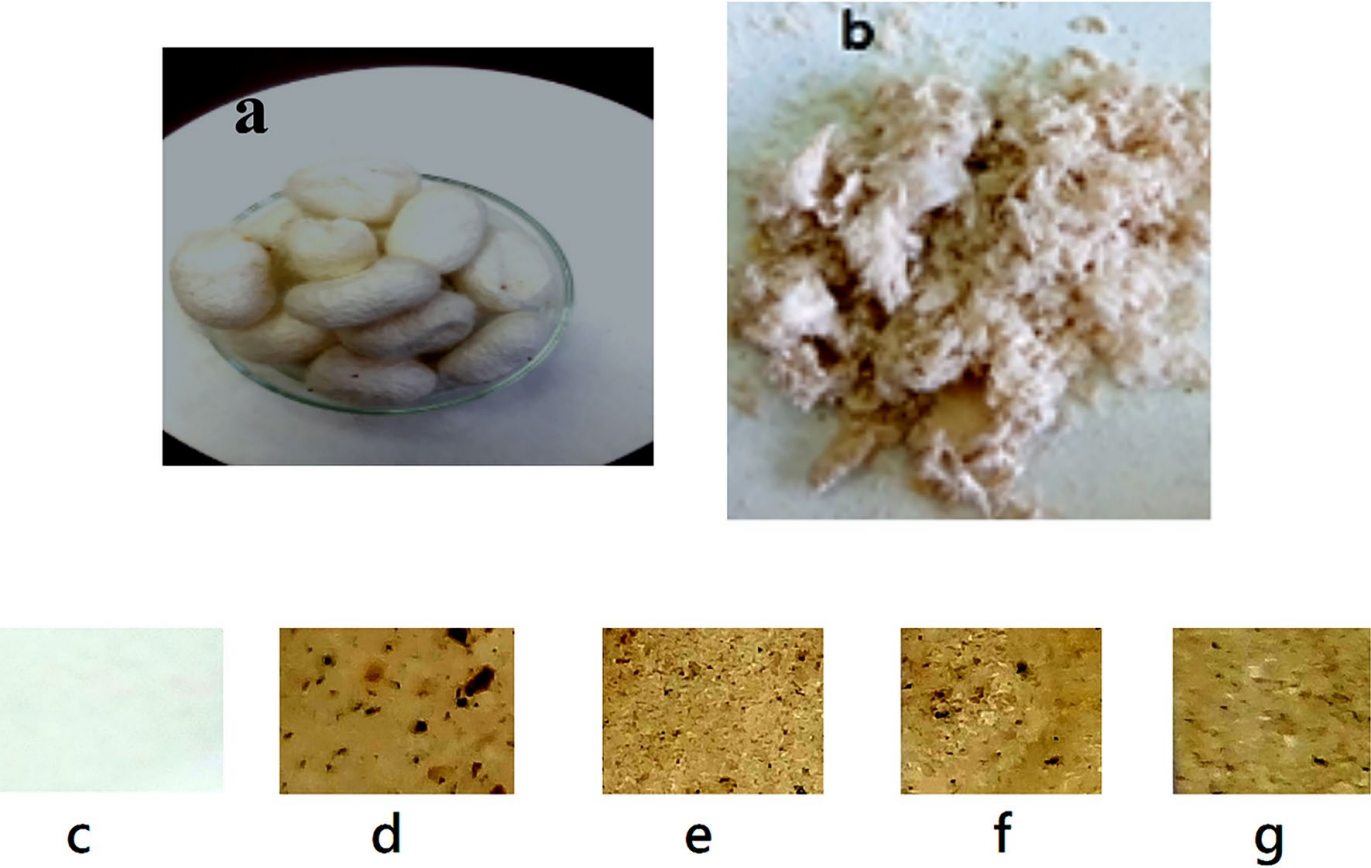
Appearance of (**a**) cocoons, (**b**) treated cocoons, (**c**) PVC, (**d**) PVC/SCW, (**e**)–(**g**). PVC/SCW/MSO Composites with MSO content 1, 2 and 3% respectively.

Figure 1a represents the whole cocoons, Fig. 1b: the cocoons after treatment, As seen in the figure, they resemble somewhat haphazard cotton fibers. PVC film (Fig. 1c) appears as transparent, smooth and homogeneous. PVC/SCW film (Fig. 1d) after mixing PVC with SCW the film became opaque, rigid, with marble like color and texture, Figures e–g after plasticizing with MSO, the marble color of the composite’s films became weaker. Because of the plasticizing effect of moringa oil, the composite became less rigid and more flexible as the amount of Moringa oil increases. The oil permeates into the polymer matrix, the components may interact more easily, potentially resulting in a smoother surface.

### Scanning electron microscope (SEM)

**Fig. 2:**
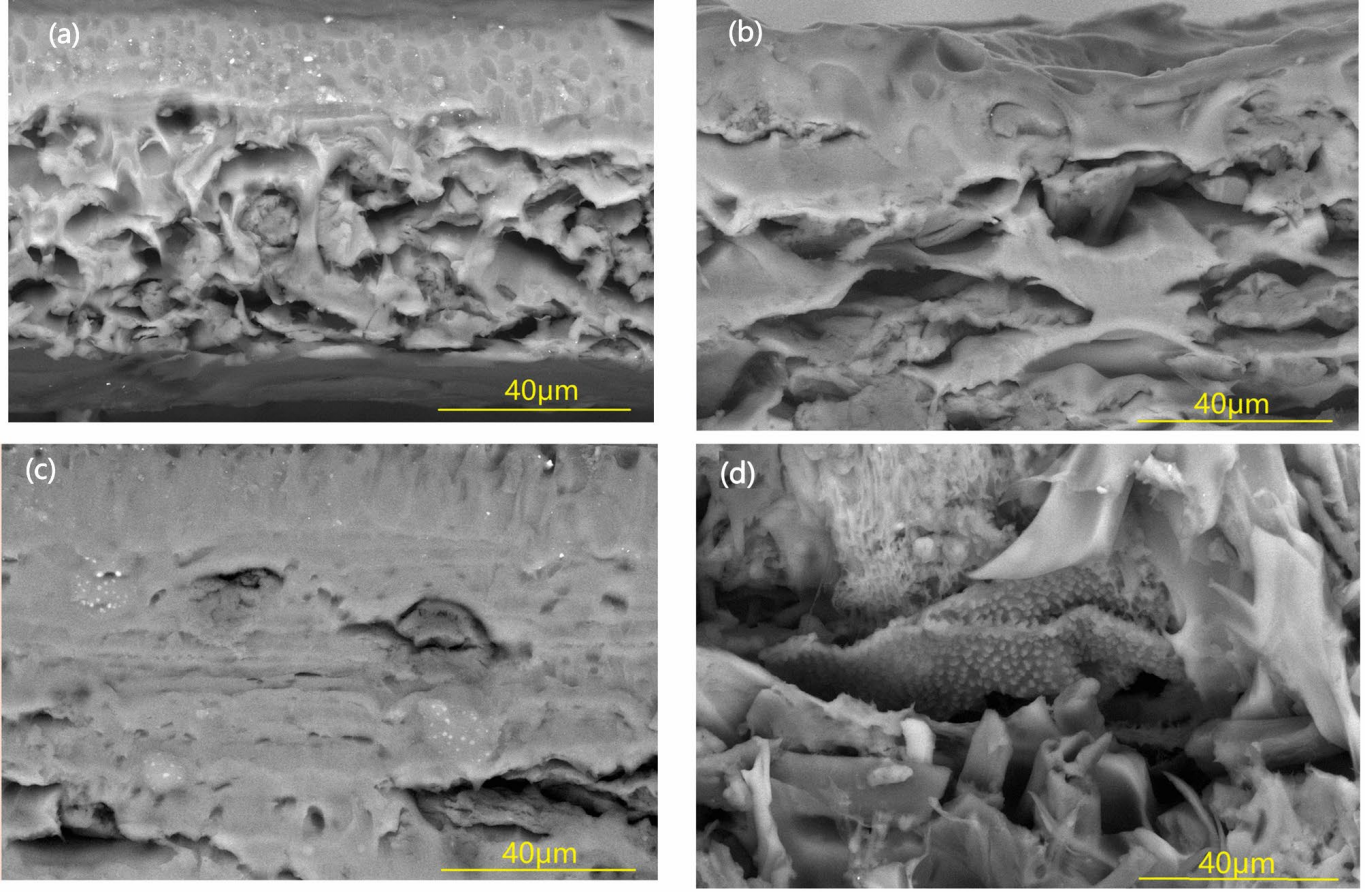
SEM images for a cross-section of (**a**) PVC/SCW, (**b**) PVC/SCW/MSO1%, (**c**) PVC/SCW/MSO 2%, and (**d**) PVC/SCW/MSO 3% [Magnification 3000X].

SEM images of the PVC/SCW cross- section and their composites with MSO are represented in Fig. 2. The cross-section of the PVC/SCW membrane in Fig. 2a reveals an irregular structure. Generally, the membrane exhibits an asymmetrical configuration with two distinct layers: an upper layer predominantly composed of silk cocoons, and a second layer containing voids and cracks. After the incorporation of MSO, these voids almost closed as seen in Fig. 2b and c. The membranes were more homogenized at MSO 2% as seen in Fig. 2c. Good oil dispersion and miscibility between phases were observed, resulting in a smooth fracture surface.

Increasing the percentage of oil to 3% (Fig. 2d) resulted in the appearance of voids, particles and discontinuous surface of the composite membrane. These were signs of immiscibility between the phases at that oil ratio.

### Fourier-transform infrared (FTIR)

**Fig. 3:**
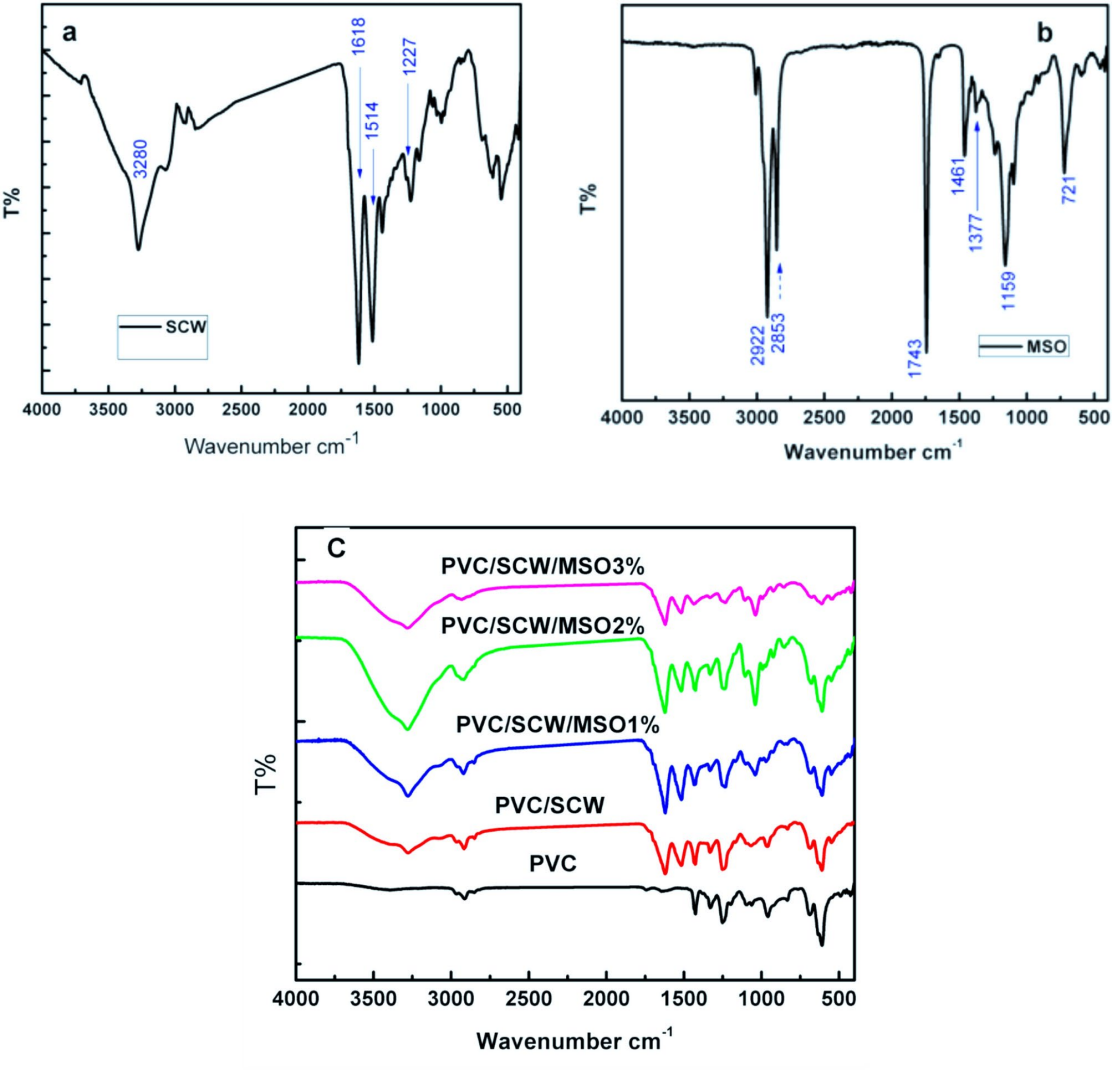
FTIR spectra for (**a**) SCW, (**b**) MSO, and (**c**) PVC &PVC/SCW/MSO composites.

FTIR spectra of the SCW, MSO, and PVC/SCW/MSO composites were recorded and displayed in Fig. 3. The FTIR spectrum of SCW (Fig. 3a) shows the protein characteristic bands, 1618 cm^−1^: C=O stretching of amide I. 1514 cm^−1^: N–H bending of amide II. 1227.88 cm^−1^ and 1444 cm^−1^: C–N stretching of amide III. 690 cm^−1^: amide IV^25,26^.

PVC spectrum (Fig. 3c) revealed the presence of a 2950 cm^−1^ peak referring to CH stretching from CH–CL, 2913 cm^−1^ corresponding to C–H stretching from CH_2_, 2860 cm^−1^ C–H_2_ stretching. 1425.69 cm^−1^ CH_2_ wagging, 1329.42 cm^−1^ due to CH_2_ deformation, 1255 cm^−1^ due to CH stretching, 957.32, 1062.89, 1197.75 and 684, 609 cm^−1^ C–Cl stretching vibration. The results obtained are consistent with the FTIR findings from other investigations^27^.

In the FTIR spectrum of the PVC/SCW, besides the PVC characteristic bands the spectrum shows the amide bands at 1620 and 1515 cm^−1^ which confirms the formation of the PVC/SCW composite.

Figure 3b represents the FTIR spectrum of moringa oil. As indicated in the spectrum, the peaks at 2922 cm^−1^ and 2853 cm^−1^ are attributed to symmetrical and asymmetrical stretching of the C–H group of CH_2_ found in fatty acids.

The stretching of the C–O bonds of the ester functional groups of lipids and fatty acids is represented by the sharp peak at 1743.58 cm^−1^.The presence of an aromatic C=C bond is shown by the peak at 1461.24 cm^−1^. At 1377.04 cm^−1^ OH bending vibration of phenols. The 720 cm^−1^ peak results from asymmetric deformation of the CH_2_ group, whereas the 1159 cm^−1^ peak is ascribed to stretching of diacylglycerol esters.

The spectrum appears comparable to that which has already been published^28,29^.

The spectra of the PVC/SCW/MSO composites (Fig. 3C) indicated that, most of the MSO peaks are primarily masked by the peaks of the PVC/SCW composites. MSO only affects the intensity of the composite peaks. An increase in peak intensities at 3278, 1618, 1041, and 610 cm⁻^1^ was observed up to MSO concentration of 2%, followed by a decrease in the oil ratio of 3wt %. It seems that MSO acts as not only a plasticizer but also a compatibilizer that improves the dispersion of the SCW into the polymer matrix leading to better alignment and enhanced detection of certain molecular vibrations. While increasing MSO content to 3% might cause phase separation which reduces the interaction between oil and matrix which lowering the peaks intensities.

### Dielectric measurements

**Fig. 4:**
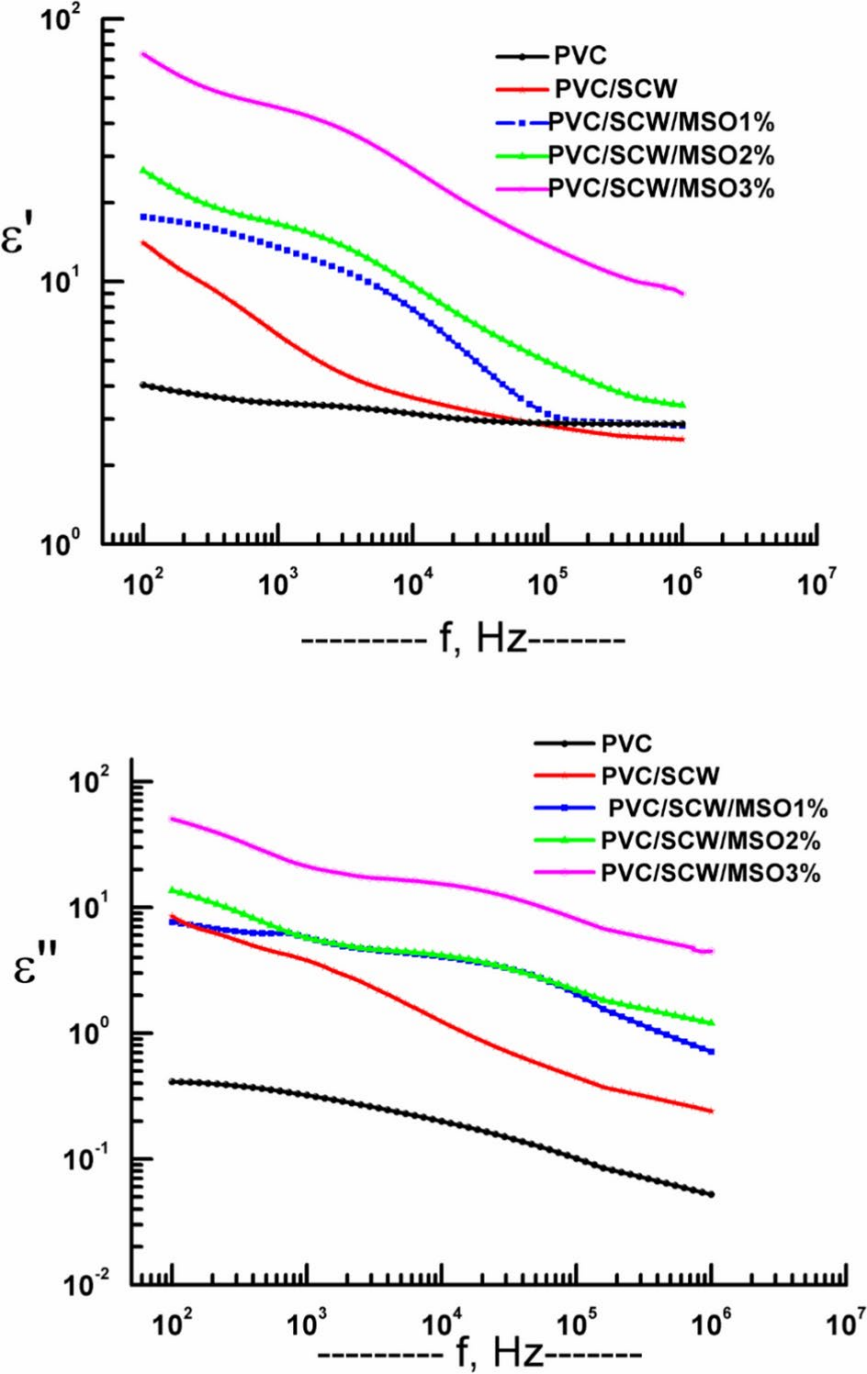
ε′ and ε″ versus the applied f, for the composites under investigation.

Ε′ and ε″ as a function of the applied f, for PVC and PVC/ SCW 50:50 wt/wt were measured and given in Fig. 4. This figure shows that both values were more pronounced, especially at low f. This finding may be due to the protein nature of WSC, which possesses polar groups in its structure responsible for the rise in both ε′ and ε″. Also, both values of ε′ and ε″ are comparable with those listed in the literature^30–32^.

Figure 4 Also depicts the ε′ and ε″ versus f for PVC/SCW after adding three percentage of MSO (1, 2, and 3%) as a plasticizer and antimicrobial agent as well^11,33,34^.

**Fig. 5:**
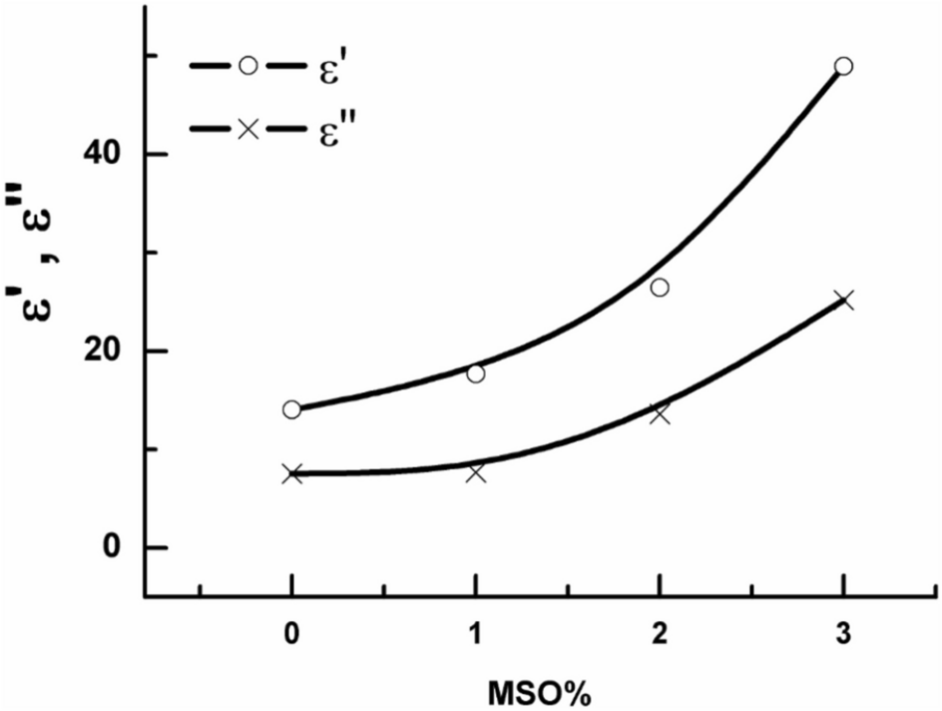
ε′ and ε″ versus the MSO content at f = 100 Hz.

From the graphs given in Fig. 4 both ε′ and ε″ values increase by increasing the content of MSO in the composite. To understand the behaviour of ε′ and ε″, both values were plotted graphically in Fig. 5 at a fixed frequency f = 100 Hz versus MSO content. From this figure, it could be concluded that both ε′ and ε″ increase by increasing MSO content, and the composite that contains 3% shows the highest value of both. This logic finding was due to the flexibility of the sample that was noticed by the increasing of the MSO content in one hand and on the other hand both values increased because of the addition of polar material (MSO) which add polarity under the influence of the electric field and consequently both ε′ and ε″ increased as seen.

On the other hand, Fig. 4 reveals that ε′ decrease by increasing f, showing anomalous dispersion that is usually appears in most polymeric composites^35,36^.

The behaviour of ε″ versus the applied frequency is broad as usual and possesses more than relaxation process in addition to the losses that come from the electrical dc conductivity.

**Fig. 6:**
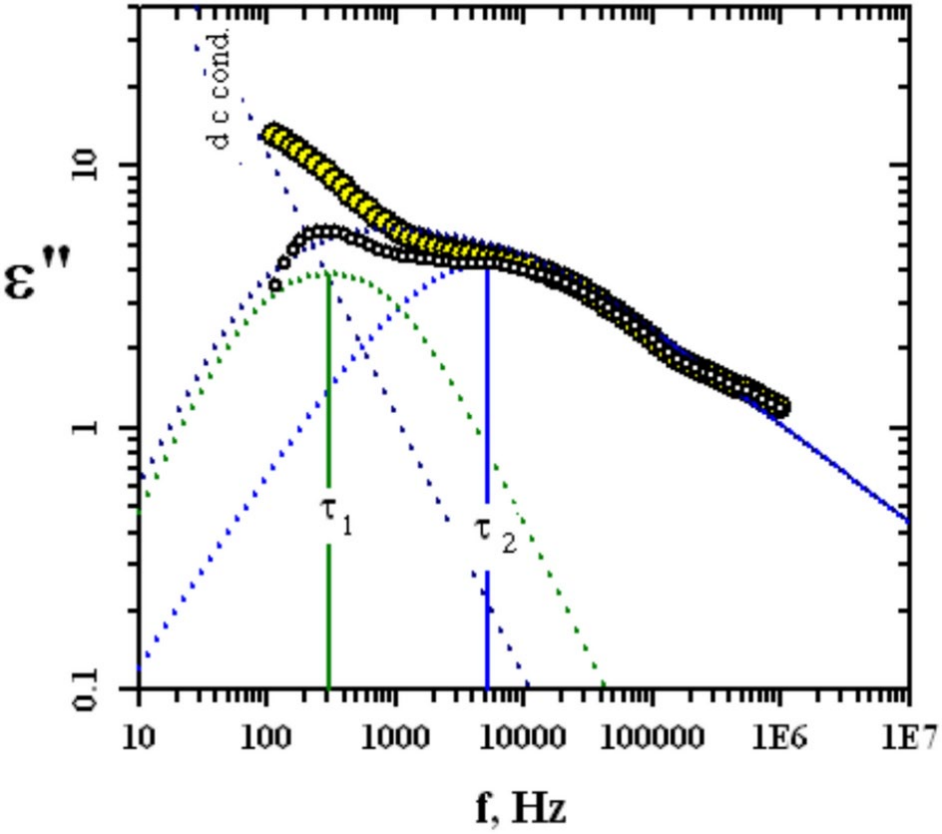
Example of the data analysis for PVC/SCW/MSO 3%

For that reason, the dc losses were subtracted and the rest of the losses were analysed based upon Frohlich term with distribution parameter *P* = 3 and Havriliak Negami function with CC = 0.25 and CD = 0.5 based on the formulae provided elsewhere^37^. Model of the analysis for PVC/SCW/MSO 3% was given in Fig. 6. The first process at a lower frequency with relaxation time τ_1_ in the order of 0.0004–0.0005 s was ascribed the interfacial polarization and or / Maxwell Wagner effect that usually appear in that range of frequency for composites that consists of different materials each of it possesses different permittivity and conductivity^10^. This relaxation process was not affected by the content of MSO. The second one, τ_2_ in the order of 10^–5^ s found to increase by increasing the MSO content. Due to the presence of oil the molar volume of the rotating unit increased and consequently the relaxation time. This is because of the aggregation of oil droplets which hindered the motion of the system and consequently the relaxation time increased.

Anyhow, the values of both ε′ and ε″ obtained for such composites may suggest the end product which possess high k values to act as energy storage materials^38^.

The dc electrical conductivity was calculated from the measured resistance using the relation σ = l/RA where R is the resistance and l is the sample thickness and A is its area. The values of σ also obtained from the analysis and found to be comparable with that measured.

**Fig. 7:**
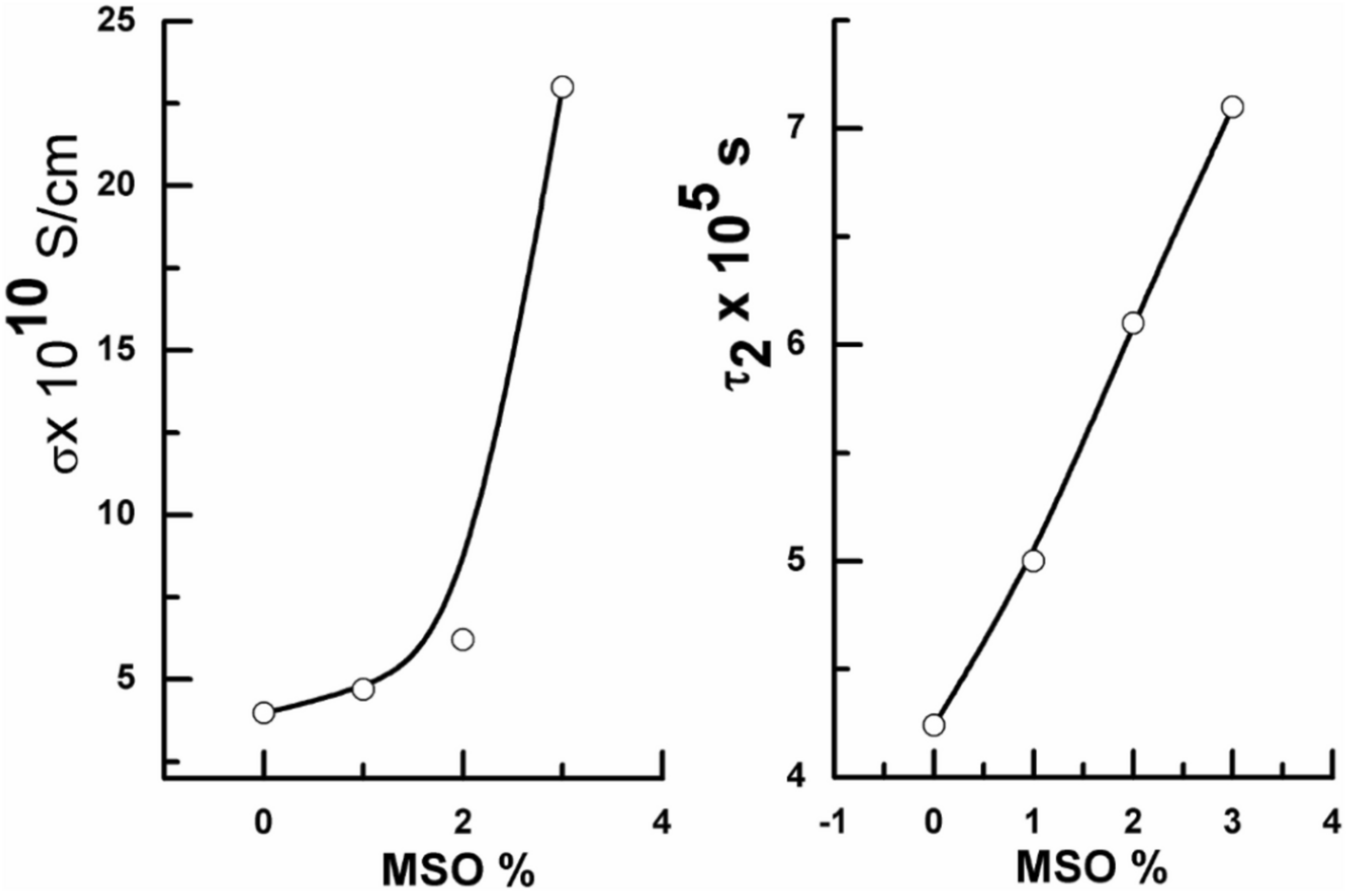
The variation of both σ_dc_ and τ_2_ versus MSO content.

The obtained data of both σ _dc_ and τ_2_ was illustrated graphically versus MSO content in Fig. 7.

σ_dc_ values are in the order of 10^–10^ S/cm which extremely suggested these composites for anti-static applications. For such applications the required range is 10^-^^9^–10^-^^14^ S/cm^11^.

### Contact angle measurements

One important metric for measuring a surface’s wettability and determining its affinity for water is the water contact angle (WCA). Table1 displays the static WCA values and appearance for PVC and its composites.

**Table 1:** Contact angle values and images of a water droplet for PVC and PVC/SCW/MSO composites.

Samples	Contact angle (°) X ± SD	Images of a water droplet on the surfaces
PVC	75.25 ± 4.99	
PVC/SCW	72.43 ± 2.4	
PVC/SCW/MSO 1%	68.73 ± 1.855	
PVC/SCW/MSO 2%	59.23 ± 3.808	
PVC/SCW/MSO 3%	81.17 ± 2.02	

X average, ± SD standard deviation

As seen the WCA for pure PVC film was 75.25 ± 4.99, which is comparable with that previously reported^39^. These values decreased to 72.43 ± 2.4 for PVC/SCW, and this may be due to the changes in the surface chemistry of PVC/SCW compared to the pure PVC. As indicated by the morphological appearance and SEM images increasing cavities and roughness have been observed; rough surfaces have a bigger total surface area than smooth ones because of the small depressions and thus offer a larger wetting area^40^. For samples containing MSO, 1 and 2%, the WCA values show a decreasing trend to become 68.73 ± 1.855 and 59.23 ± 3.808 ,respectively. Decreasing WCA after incorporation of oil into the samples may be explained by the existence of phenol compounds on the sample surface, which possibly raise the interaction with water drops. It also can be a result of adding polar groups to the film’s surface.

This decrease is followed by an unexpected increase in higher oil percentage to reach 81.17 ± 2.02 at 3% MSO content which may be explained by increasing the oil’s plasticizing impact, which disrupts the film’s network structure. Similar behavior was previously reported^41,42^.

### Thermogravimetric analysis (TGA)

The TGA technique is crucial for researching the thermal characteristics of materials. It gives information about the degradation patterns, thermal stability ,moisture content of the material and residual inorganic materials.

**Fig. 8:**
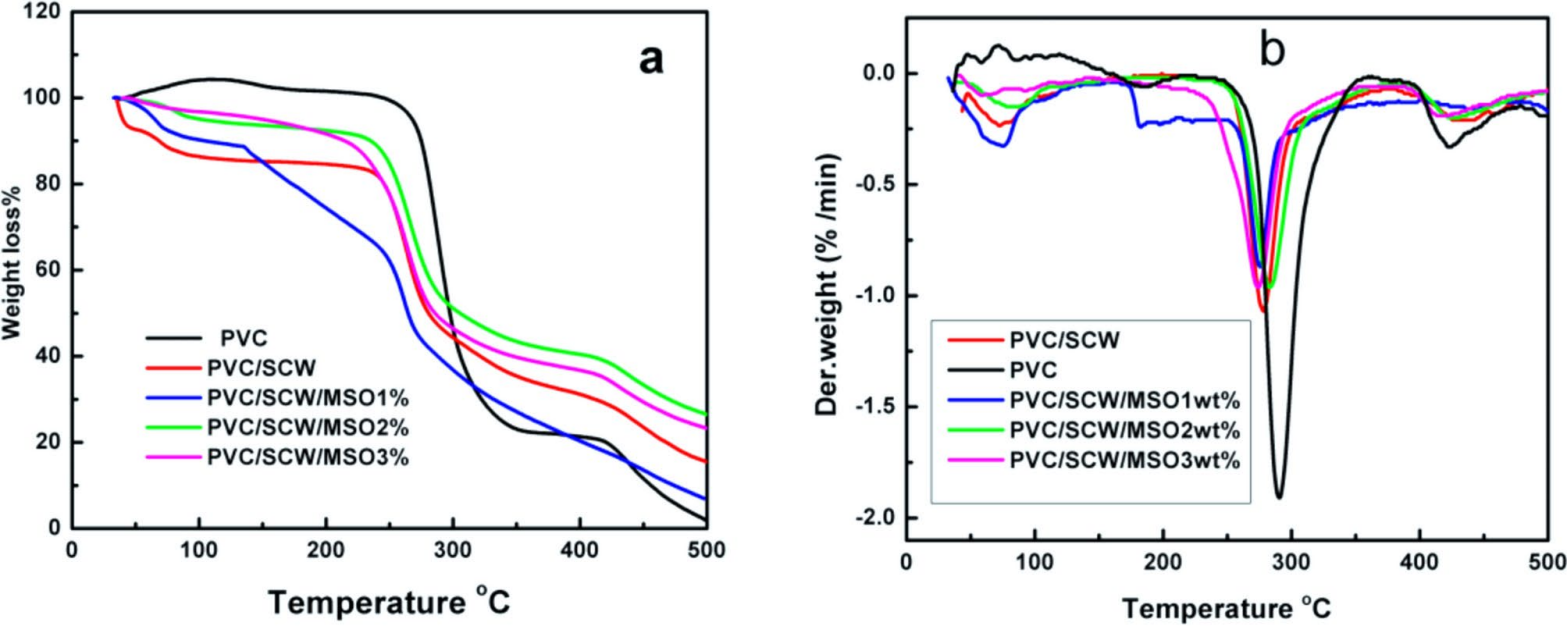
(**a**) TGA and (**b**) Derivatives for PVC, PVC/SCW, and PVC/SCW/MSO with different concentrations of MSO.

**Fig. 9:**
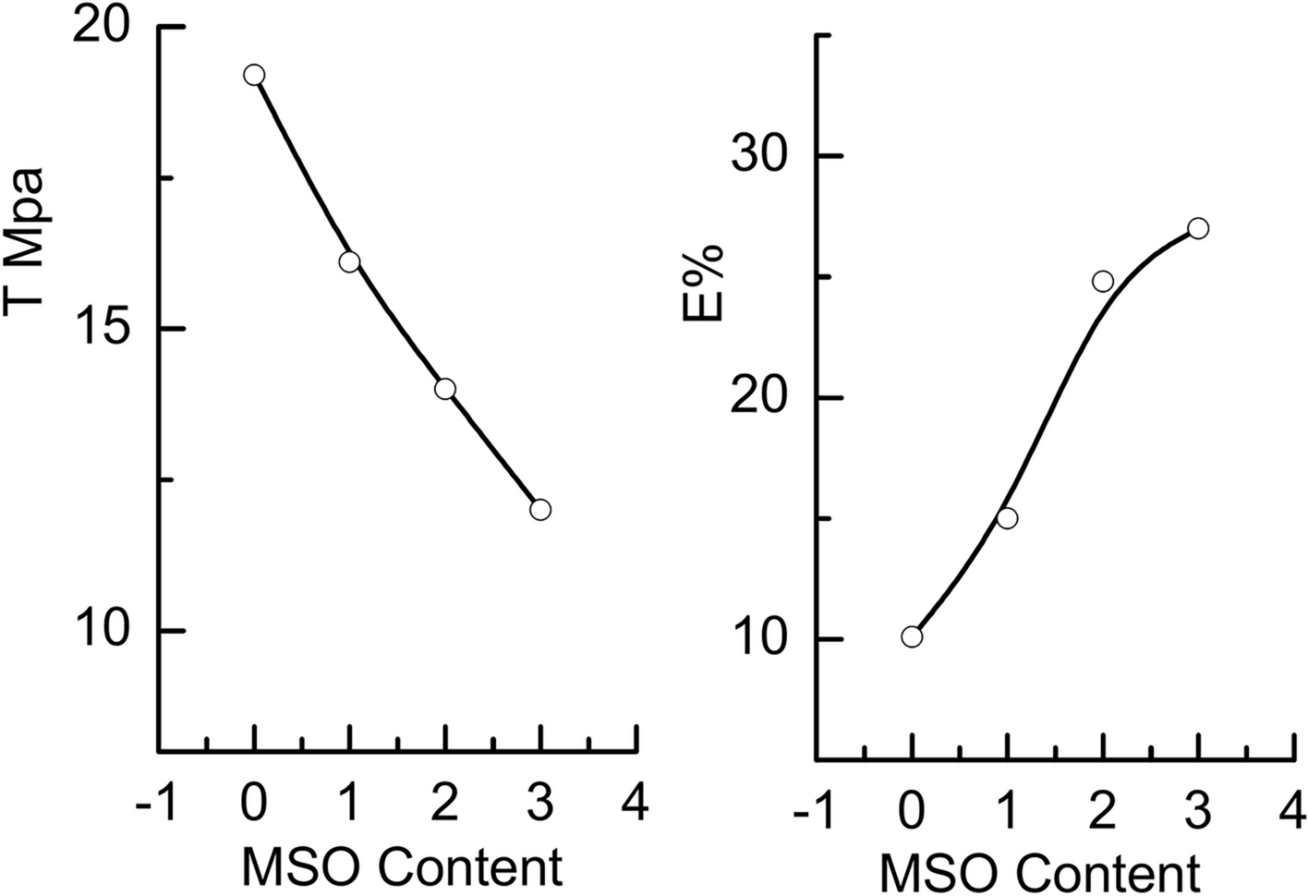
Tensile strength and elongation at break for PVC/SCW/MSO composites versus MSO content.

Thermogravimetric curves for neat PVC, PVC/SCW and PVC/SCW/MSO composites are given in Fig. 8a and the corresponding derevatographs are represented in Fig. 8b. The degradation parameters of the decomposition processes are listed in Table 2 (Fig. 9).

**Table 2:** Thermal decomposition parameters for PVC, PVC/SCW and PVC/SCW/MSO composites.

	Temperature at characteristic weight loss (°C)				
	T_onset_	T_deg_	T_10_	T_50_	Residual mass at 500 °C (%)
PVC	270	292	274.9	297	0
PVC/SCW	203	278	68.84	278	16.16
PVC/SCW/MSO1%	95	275.76	105.7	264.7	7.11
PVC/SCW/MSO2%	227	282.75	235.5	305.34	28.85
PVC/SCW/MSO3%	197.5	273.31	215.55	285.55	23.5

T_10_: The temperature at 10% Weight Loss (°C).

T_50_: The temperature at 50% Weight Loss (°C).

T_deg_: the temperature of maximum degradation (°C).

T_onset:_ The temperature at which the transition begins (°C).

There are two primary phases in the thermal decomposition of PVC. The first process, dehydrochlorination, removes hydrogen chloride, leaving behind unsaturated hydrocarbons with long conjugated double bonds. In the second stage, the hydrocarbon backbone is broken down by hydrogen being subtracted from it by chlorine free radicals, producing HCl gas as a byproduct^43^.

Comparing the thermogram of PVC before and after adding the treated silk cocoons, it is found that, PVC film degraded by 10% and 50% at temperatures 274.9 °C and 297 °C, respectively. For PVC/SCW film, these values were 68.84 and 278 °C respectively. It is also noticed that, the residual weight increased for PVC/SCW compared to native PVC which was comparable with another study on polyurethane-silk cocoons composites^44^.

In the PVC/SCW derivative curve, there are 3 peaks. The onset temperature shifted to a lower temperature. Derivatograph of pure PVC film exhibited 2 peaks: T_max1_ at 292 °C and (T_max2_) at 424 °C. While for PVC/SCW sample the peaks appeared at T_max1_ = 75, T_max2_ = 278 °C and T_max3_ = 434 °C.

The degradation temperature of the PVC/SCW was shifted downwards. These changes have resulted in a decrease in the thermal stability of the PVC/SCW composite. This may be due to the poor compatibility between the 2 components which are clearly seen as two phases in the SEM micrographs.

After plasticizing with MSO, the derevatographs of the composites reveal the presence of 3 peaks. The initial weight loss of about (40–120) is due to the evaporation of moisture content present in the samples. PVC/SCW/MSO1% exhibits the lowest onset degradation temperature (95 °C) which revealed poor interaction between the phases. This could result in defects or regions in which degradation takes place more rapidly.

PVC/SCW/MSO2% sample has 10% and 50% degradation at temperatures about 235.34 and 305.34 °C respectively. T_deg_ was about 282.75 °C. The residual mass was maximum at MSO2%. The results may indicate that, addition of MSO at this oil concentration (2%) introduces new bonds between PVC and SCW and suggests improved thermal stability of composites compared to PVC/SCW which was confirmed by the other characterization tools. Increasing the MSO content to 3% increases softening and the free volume in the matrix ,which makes the composite undergo degradation at a lower temperature.

### Mechanical analysis

The mechanical properties of the polymeric matrix in the presence of oil in general are very complicated and depend upon many factors, one of them is the role that such oil plays and how it affects that matrix whether it acts as a plasticizer or as a reinforcement agent.

In our case and as it is clear from the decrease in the relaxation time τ_2_ that reflects the flexibility of the matrix due to the plasticizing effect of MSO. In addition, the presence of MSO improved of the compatibility between the PVC and SCW, see the scanning electron microscopy part. Good compatibility can lead to better plasticization which improves the mechanical properties.Figure9 represents the change in tensile strength and elongation at break for PVC/SCW/MSO composites versus MSO content. As seen,

MSO can act as a plasticizer which increases the flexibility and ductility of the PVC/SCW blend. This can lead to a decrease in tensile strength (T), as the blend becomes more susceptible to deformation under stress. This behavior is consistent with other previously reported^45^. This decrease is much more pronounced up to 2% after which it becomes moderate at 3%. The values of tensile strength of the composites are suitable for lightweight fabric privacy curtains with moderate durability which usually range from 10 to 25 MPa provided by manufacturer specifications.

On the other hand, the E at break shows a noticeable increase which is unpronounced after 2%. This may be attributed to the spread of MSO in the polymeric matrix which causes some sort of elasticity and consequently an increase in E%. The addition of MSO greatly improved the mechanical properties in PVC/SCW and gave more flexibility to the blend films and the optimum concentration of MSO was 2%, after this concentration saturation occurred. From these results, it seems that the concentration of 2% of MSO provides a balanced increase in free volume (for flexibility) while maintaining sufficient interactions between polymers to sustain strength. However, oil concentrations as low as 1% may not be sufficient to improve chain mobility, making the composite rigid and excess oil concentration (3%) may disrupt the cohesive network of the polymer chains, which would result in a decrease in strength.

### Antimicrobial analysis

The antimicrobial activity of the PVC/SCW film and the films after the addition of MSO were tested against *E. coli*, *S. aureus* and *C. albicans*; the results are summarized in Table 3. The PVC/SCW film exhibits activity against all the tested strains. This antimicrobial effect is mainly attributed to the presence of silk cocoons. As mentioned before, the silk cocoon functions as a shield for the pupa from pathogens and predators. Right now, besides sericin and fibroin, a large number of antimicrobial proteins have been identified in silk cocoons as seroins and protease inhibitors^46^. These proteins exhibit wide-ranging antibacterial activity against viruses, bacteria, and fungi^47^. The antibacterial mechanism of silkworm seroins involves the binding of the C-terminal domain to bacterial peptidoglycan and the inhibition of bacterial growth by the N-terminal domain^48^.The cocoon also contains minor levels of non-protein antibacterial components such as organic acids, flavonoids, alkaloids, and heterocyclic compounds.^49^. The bacteriostatic activity of the cocoons against *S. aureus* and *E. coli* was demonstrated by Zhou, Bin, and Huiling Wang; however, the antibacterial effect against *E. coli* was more significant than that against *S. aureus*^12^.

It is noticed that plasticization with MSO adds more value to the antimicrobial action, the CFU values increased by increasing the oil content as seen in Table 3. Similar behavior was observed elsewhere^50^. The antimicrobial compounds in Moringa oil are more likely to diffuse from the composite as the oil content rises, strengthening the inhibitory action against bacteria development.

As mentioned before, Moringa oil is rich in bioactive compounds such as oleic acid, flavonoids and benzyl isothiocyanate^51.^ These compounds exhibit antimicrobial properties that can disturb bacterial cell membranes leading to leakage of intracellular contents and cell death. Fungi, such as *C. albicans*, which are responsible for infections, are also sensitive to MSO. Higher oil concentration in the composite increases the oil’s effectiveness in disrupting fungal cell walls or interfering with the cellular processes.

Furthermore, the surface structure is altered by the addition of moringa oil. These modifications to the surface can stop biofilm formation, a major problem in microbial infection, and make it resistant to microbial adhesion.

**Table 3:** (%) CFU reduction of pathogenic strains for PVC/SCW/MSO composites.

	CFU reduction %		
Sample	*E. coli*	*S. aureus*	*C.albicans*
PVC/SCW	48.16	41.87	33.19
PVC/SCW/MSO1%	69.72	62.71	67.56
PVC/SCW/MSO2%	72.49	79.83	77.55
PVC/SCW/MSO3%	82.43	83.54	81.81

Various studies have noted variations in the antibacterial activity of MSO seed oil; these variations may be related to variations in moringa species and the techniques they employed to obtain oil^52^. It is worth mentioning that, despite increasing the hydrophilic nature of films by the addition of MSO up to 2% as discussed before, the films exhibited improved antimicrobial behavior. Whereas hydrophilic surfaces are likely to facilitate speedier bacterial adherence because the hydrophilic characteristics of the polymer surface aid in the bacterial cells’ faster attachment. Simply, in fact, the composite has an intermediate surface that is neither highly hydrophobic nor highly hydrophilic. These types of surfaces can exhibit mixed properties, where both microbial attachment and antimicrobial agent release are moderated. In addition, there are many other factors of material properties, such as surface charges, and material topography, that also affect bacterial and fungal attachment to surfaces^53,54^.

## Conclusion

New antimicrobial PVC films were prepared by adding MSO to a PVC/SCW polymer composite. The neat PVC film was transparent, and the transparency decreased with the addition of SCW and MSO. As shown in electron microscopy images, the PVC/SCW film exhibited a heterogenic structure. After addition of MSO 2% the films were more homogenized. Blending the composites with MSO significantly improved the dielectric properties of the composite films. The antimicrobial activity of the composites increases by increasing the MSO content. The hydrophilicity increases with the addition of up to 2% MSO. Moringa oil as additive at a certain concentration interacts with both SCW and the PVC, influencing the overall thermal degradation behavior and increasing the compatibility between the two components and acting as a compatibilizer. The improved properties of the PVC/SCW/MSO2% composites nominated them for effective use as antimicrobial hospital curtains and sheets.

## Data Availability

All data generated or analyzed during this study are included in this published article.
